# GM-CSF co-expressing DNA/MVA vaccine, prevention of acquisition by two series of SIVE660 challenges followed by a series of SIV251 challenges

**DOI:** 10.1186/1742-4690-9-S2-P25

**Published:** 2012-09-13

**Authors:** H Robinson, S Kannanganat, S Gangadhara, L Lai, T Yu, P Kozlowski, P Earl, B Moss, RR Amara

**Affiliations:** 1 GeoVax Inc., Smyrna, GA, USA; 2 Emory University, Atlanta, GA, USA; 3 Louisiana State University Health Sciences Center, New Orleans, LA, USA; 4 National Institute of Allergy and Infectious Diseases, Bethesda, MD, USA

## Background

In 2010 we reported prevention of acquisition of a repeated SIVE660 challenge in rhesus macaques vaccinated with a SIV239 DNA/MVA vaccine that co-expressed GM-CSF and VLP in the DNA prime. The reduced risk of infection correlated with the avidity of Env-specific IgG. Here we report studies on the longevity and breadth of this protective response.

## Methods

Following the initial 12 challenges, 5 uninfected rhesus were monitored for one year, boosted with 1x10^8^ pfu of MVA/SIV239, and re-challenged 6 months later with 12 weekly rectal doses of SIVE660. The resulting four uninfected macaques were held an additional 6 months and challenged with 12 weekly rectal doses of SIV251. Avidity of Env-specific IgG was determined using a NaSCN elution ELISA. Per exposure efficacy was estimated using a leaky effects model.

## Results

Per exposure efficacies were 90% and 94% for the 1^st^ and 2nd SIVE660 series, respectively, and 72% for the SIV251 series of challenges. For the SIVE660 series, 50% infection was reached by the 3^rd^ challenge for controls, but never reached in vaccinated animals. For the SIV251 series, 50% infection was reached by the 2^nd^ challenge for controls but not until 10 challenges for vaccinated animals. Both SIVE660 and SIV251 series showed transient low “blips” of virus. None of five E660 “blips” resulted in anamnestic systemic Ab, whereas two of three SIV251 blips resulted in such. Correlates also differed for the two infections with the avidity of Env-specific IgG correlating with prevention of acquisition for SIVE660 but not SIV251.

## Conclusion

A DNA/MVA vaccine in which GM-CSF is co-expressed in the DNA prime can provide substantial prevention of acquisition against serial challenges over a three year period of time. Our results also reveal SIVE660 and SIV251 rectal challenges differing in their ability to initiate systemic Ab responses and in their correlate for prevention of acquisition.

